# Gender-specific differences in valvular heart disease

**DOI:** 10.1007/s00508-019-01603-x

**Published:** 2020-01-29

**Authors:** Christian Nitsche, Matthias Koschutnik, Andreas Kammerlander, Christian Hengstenberg, Julia Mascherbauer

**Affiliations:** Department of Internal Medicine II, Medical University of Vienna, Waehringer Guertel 18–20, 1090 Vienna, Austria

**Keywords:** Aortic stenosis, Mitral regurgitation, Tricuspid regurgitation, Sex, Women

## Abstract

The development of new devices and treatment options has greatly increased the interest in heart valve diseases. In this context, the consideration of gender differences in diagnosis, treatment success, and prognosis is of great importance. Available data show that women and men with heart valve disease have different risk profiles, which have a significant impact on treatment outcomes and prognosis.

It is the purpose of this review article to give an overview of gender-related differences in patients with valvular heart disease, regarding clinical presentation, treatment, and outcomes. In light of the emerging treatment possibilities, future research should emphasize the role of gender since both sexes benefit from tailored management.

## Introduction

The understanding and management of valvular heart disease has recently seen groundbreaking progress. We have not only come to refine our pathophysiological insights, but the development of new therapeutic opportunities now enables us to offer treatment to patients formerly deemed inoperable. In light of these major therapeutic advances, an exact patient characterization, including sex-specific differences, is pivotal to provide optimal treatment. This overview focuses on sex-related differences in patients with valvular heart disease regarding patient presentation, treatment, and outcomes.

## Aortic valve

### Aortic stenosis

**Fig. 1:**
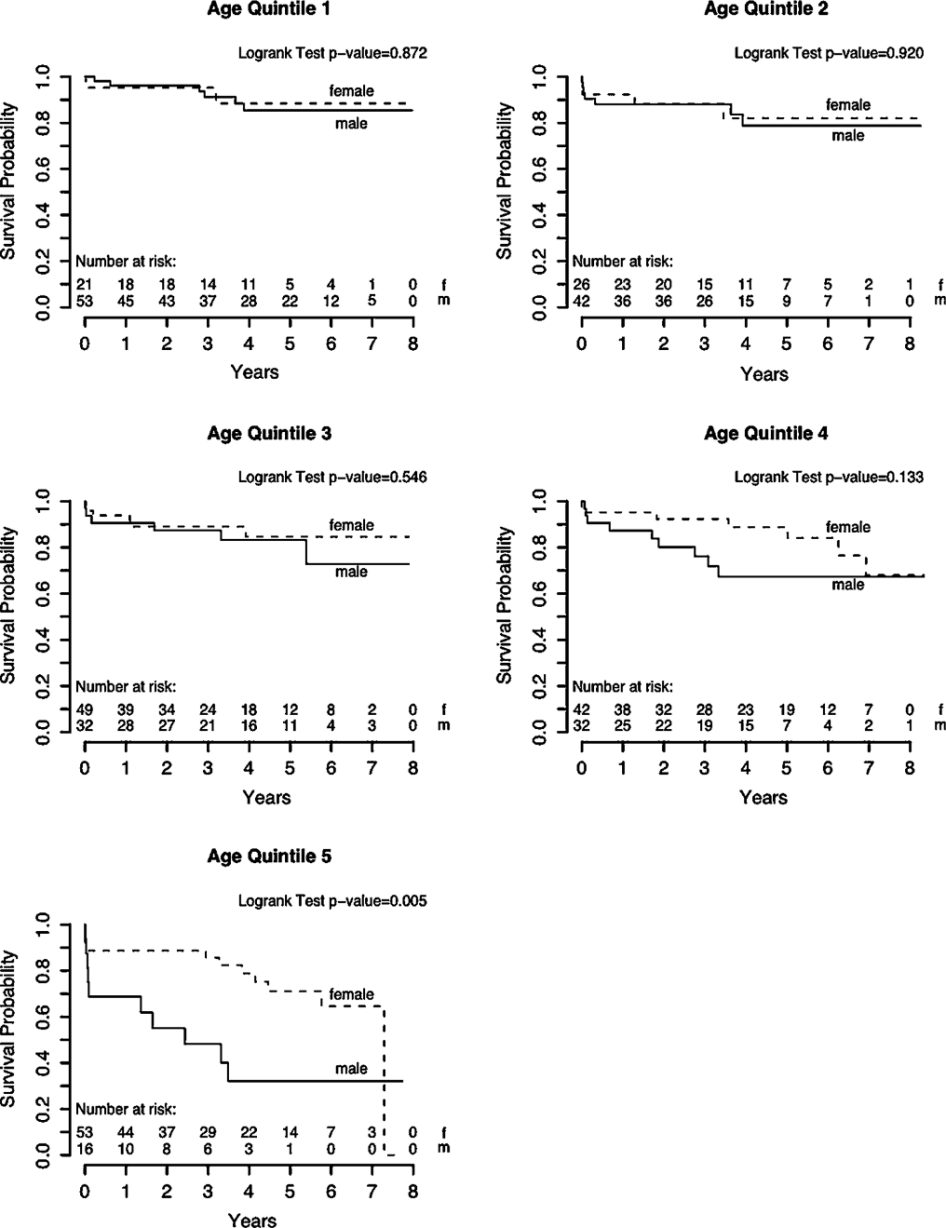
Outcomes following surgical aortic valve replacement. Survival rates after surgical aortic valve replacement are comparable between sexes across age groups apart from the fifth age quintile (78 years or older), where women showed superior survival as compared to men. (Figure printed with permission from Fuchs et al. [7])

**Fig. 2:**
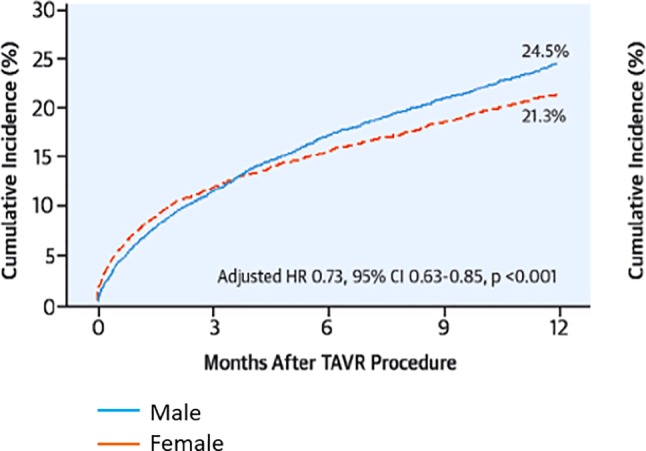
Differences in outcomes following transcatheter aortic valve replacement. Women with severe aortic stenosis (AS) experience lower mortality rates compared to men following trans-catheter aortic valve replacement (TAVR). *HR* hazard ratio, *CI* confidence interval. (Figure printed with permission from Chandrasekhar et al. [14])

Due to the aging population, aortic stenosis (AS) represents the most common indication for valve replacement in Europe, with rapidly increasing prevalence [1]. Degenerative aortic valve calcification (AVC) is the major mechanism of AS. The degree of calcification on multidetector computer tomography (CT) scans has been demonstrated to reflect AS severity, irrespective of sex; however, women display a steeper slope of AS severity increase with any given increase of AVC load than men. The biological background for this phenomenon is still unclear but might be due to differential importance of AVC-promoting factors in women and men, such as vitamin D receptors and growth factors [2]. The presence of severe AVC was shown to predict worse prognosis in AS, however, it holds similar survival implications for both sexes [3]. Quantification of AVC by CT is of particular clinical importance in low-flow AS, if dobutamine stress echocardiography is not possible/inconclusive, and in patients with paradoxical low-flow AS. Here, gender-specific cut-off values should be applied as women reach the same hemodynamic AS severity with less AVC [4], due to more pronounced valvular fibrosis instead of calcification [5]. With the onset of cardinal symptoms, such as angina, exertional dyspnea and syncope, AS patients face a dismal prognosis and should receive valvular replacement according to current recommendations [6]. Interestingly, risk profiles of women referred for surgical aortic valve replacement (SAVR) differ substantially from those of men. Females undergoing SAVR were reported to have more severe exertional dyspnea, higher frailty scores, and more severe AS, as determined by valve area and mean pressure gradient [7, 8]. Additionally, across most studies, women scheduled for SAVR were significantly older than men [7–9]. These observed differences in age at surgery were hypothesized to derive from a lower prevalence of bicuspid aortic valves in women, from later presentation to a physician, or from later referral to surgery [7]. Moreover, the smaller body size of women entails obvious anatomical specifics that make interventions technically more demanding, frequently require the use of smaller prosthetic valves and additional aortic annular enlargement [10]. According to the elevated preoperative risk of women it is not surprising that they suffer worse postoperative short-term survival across the majority of trials assessing gender-specific outcomes [8, 9]; however, data on long-term survival following SAVR suggest no gender-related differences regarding mortality [11] or even show favorable outcomes for females ([7]; Fig. 1). This has been attributed to the longer life expectancy of women in the general population. With the increasing use of transcatheter aortic valve replacements (TAVR), the topic of gender equality has recently been revisited by numerous studies. As with SAVR, female patients referred to TAVR display a distinct risk profile. In general, women are older, present with better left ventricular function, as well as a lower prevalence of coronary artery disease, prior coronary intervention, diabetes and atrial fibrillation [12–14]. Female anatomy affects procedural characteristics for TAVR as well and may partly be held accountable for gender differences regarding the spectrum of complications. Shorter distance from the coronary ostia to the annulus and a higher prevalence of severe aortic calcification and horizontal aorta were discussed to be responsible for a higher incidence of periprocedural coronary obstruction and conversion to open surgery in women [14]. Conversely, smaller annuli in females were reported to allow better cover index and less paravalvular regurgitation [12, 14]. Large sized TAVR trials reported less frequent transfemoral access in women, which likely reflects the lack of small sheath sizes during early TAVR experience [12, 14]. Additionally, smaller peripheral vessels in women were found responsible for a higher rate of vascular complications and major bleeding, which might, however, not entail worse outcomes due to the amenability to prompt vascular management [12–14]. Overall, women undergoing TAVR face equal 30-day outcomes, but superior long-term survival compared to men, irrespective of the selected access route ([12–15]; Fig. 2). Regarding gender-related differences in terms of cost-effectiveness, available TAVR data are mostly historical [16] and do not include intermediate and low-risk patients; however, the use of TAVR seems to be particularly beneficial in female patients, an issue that shall now be targeted by future trials.

**Fig. 3:**
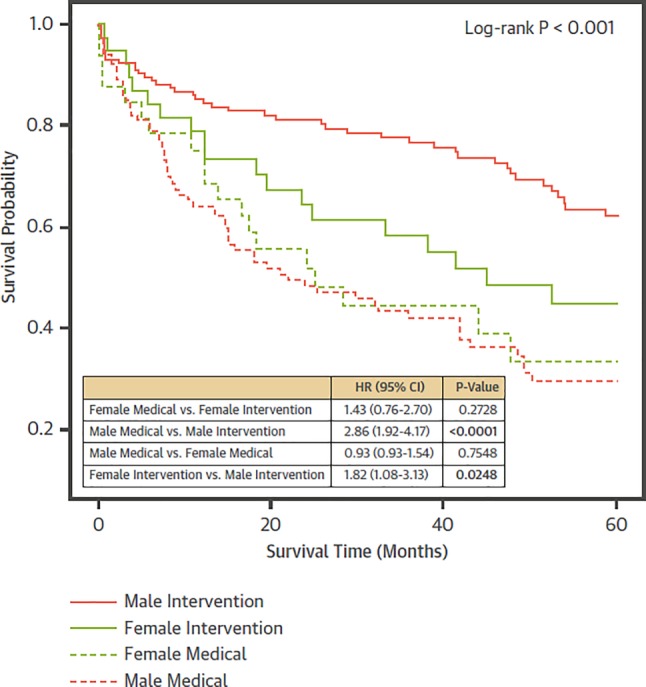
Gender-related differences in low-gradient, low-ejection fraction aortic stenosis. Women suffer worse outcomes after intervention for low-gradient, low-ejection fraction AS in comparison to men. (Figure printed with permission from Bartko et al. [17])

With respect to low-gradient, low-ejection fraction AS recent data indicate that women underreport symptoms but seem to present in more advanced stages of disease as suggested by worse functional capacity, more syncope and more eccentric left ventricular (LV) remodelling despite similar stenosis severity and less coronary artery disease. In patients with low-gradient, low-ejection fraction AS undergoing aortic valve intervention, women had a higher risk of mortality compared to men ([17]; Fig. 3).

### Aortic regurgitation

Severe aortic regurgitation should be treated as soon as symptoms occur, left ventricular function declines, or if the size of the left ventricle exceeds certain limits [6]. It has been shown that female patients were underrepresented in studies forming the basis for guideline recommendations concerning left ventricular size limits with respect to aortic regurgitation [18, 19]. As a result, these limits are mainly based on male patients who on average have a significantly larger heart [20]. Thus, women reach respective cut-off values that indicate surgery at an advanced stage of disease which may be the cause of excess mortality following treatment [21]. This has led to the assumption that correction of left ventricular diameters for body surface area (BSA) appears more suitable for the definition of cut-off values. Indeed, the use of indexed end-systolic diameter was shown to improve the prediction of adverse outcomes after surgery in patients with low BSA, but not in those with high BSA [22]. Based on this study, the indexed end-systolic diameter has been included in the guidelines as an alternative value for patients with small body size [6].

## Mitral valve

Mitral regurgitation (MR) represents the second most frequent indication for valve surgery in Europe [6]. Facing different prognosis and treatment strategies, it is important to distinguish between primary MR, where regurgitation is caused by a diseased mitral valve apparatus and secondary MR resulting from alterations of LV geometry.

### Primary MR

**Fig. 4:**
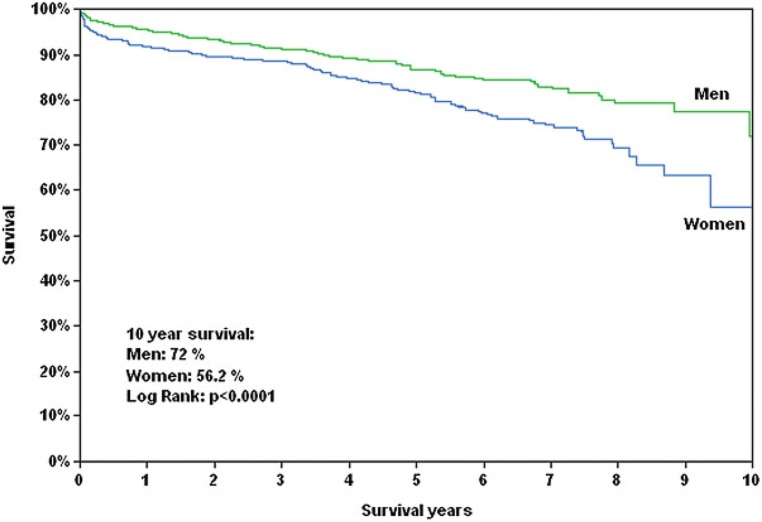
Outcomes following minimally invasive mitral valve surgery. Women experience worse long-term survival after minimally invasive mitral valve surgery as compared to men. (Figure printed with permission from Seeburger et al. [33])

In Europe, the most common cause of primary MR is valvular degeneration, including a large spectrum of lesions, ranging from simple chordal rupture that can cause mitral valve (MV) prolapse or flail leaflet, to a significantly altered myxomatous valve with excess tissue and multisegmental prolapse with or without flail leaflet [23]. The onset of heart failure symptoms as well as signs of LV dysfunction (ejection fraction of ≤60%) and LV dilatation (left ventricular end-systolic diameter ≥45 mm) in asymptomatic patients with severe primary MR represent class I indications for mitral valve repair [6], which has been shown to improve survival regardless of sex [24]. The prevalence of MV prolapse in the general population was found to be higher among women [25, 26], but men predominate in patients undergoing mitral valve surgery/intervention [24, 27, 28]. To clarify this discrepancy, it is important to address clinical, anatomical and physiological sex-related differences in primary MR. The prevalence of rheumatic MV disease as well as heart failure symptoms were reported to be higher among females [27]. Furthermore, women were reported to more often present with diffusely myxomatous valves than men, characterized by anterior and bileaflet prolapse, extensive leaflet thickening and less flail leaflets [29]. In addition, women present with smaller absolute regurgitation volumes, and smaller left atrial and ventricular dimensions compared to men when referred to surgery [29]; however, differences in cardiac dimensions as well as regurgitant volumes can be significantly reduced when values are indexed to body size [27]. In this context it is important to mention that cut-off values of cardiac dimensions indicating the need for surgery were established using predominantly male populations [30] and to this day these cut-offs are not systematically indexed to body size [6, 31]. Consequently, less women reach the recommended surgical criteria of ventricular enlargement in MR [6], which may result in worse outcomes after surgery. Data from large sized cohort studies suggest advantageous outcomes for men after MV surgery (Fig. 4); however, these trials were retrospective in design and not confined to a pure organic MR cohort [32, 33]. Moreover, female gender was related to a higher probability of recurrent heart failure after MV surgery [27]. A potentially higher increase of mean mitral valve gradient with exercise after MV repair, and a higher prevalence of diastolic dysfunction in women were hypothesized to explain these findings [34]. Conversely, one recent study showed no sex-related differences in survival following MV surgery, whereby LV remodelling, recurrence of MR and decline of pulmonary artery pressures were similar in men and women [27]. As for minimally invasive treatment strategies of primary MR, percutaneous edge to edge repair was shown to be non-inferior compared with conventional surgical MV repair with respect to 1‑year and 5‑year survival, irrespective of sex in patients deemed inoperable [28, 35].

### Secondary MR

In contrast to primary MR, the causative factor of secondary/functional MR is not intrinsic to the valve, but a result of geometrical changes of the LV due to ischemic or non-ischemic etiologies. As trials continuously failed to demonstrate a survival benefit of surgical intervention in these patients [36], current recommendations are very restrictive regarding indications for invasive treatment of secondary MR, particularly in patients not eligible for coronary revascularization [6]; however, with the emerging popularity and refinement of percutaneous repair techniques, interventional treatment of functional MR currently is a topic of intense discussion. Controversial data were recently reported in two large randomized controlled trials with respect to the outcomes after transcatheter MV repair in patients with functional MR [37, 38]. While no 2‑year survival benefit was found in patients undergoing percutaneous repair compared with optimal medical therapy in the MITRA-FR trial, the larger sized COAPT study reported a significant reduction in mortality and heart failure hospitalization after 36 months in subjects receiving MV repair with the MitraClip device. These results will certainly enhance the search for optimal treatment criteria in order to identify patients who are likely to benefit from MV repair [39]. In this context, few studies have focused on gender-related characteristics in patients receiving percutaneous edge to edge repair [40–42]. Left ventricular reverse remodelling (LVRR) following MV repair has been reported for both sexes [43] and has recently also been established as a factor of pivotal prognostic significance [42]. Interestingly, in this study female sex proved to be the strongest predictor of LVRR, followed by non-ischemic etiology of MR, freedom from heart failure hospitalization 6 months prior to intervention, absence of diabetes mellitus, and LV end-diastolic diameter <75 mm [42]. Given the prognostic value of LVRR, it seems obvious to link female sex to a higher survival probability after minimally invasive MV repair; however, data from registry-based trials reported equal results with respect to clinical outcomes and LVRR for men and women [40, 41]. These registries were, however, not confined to a pure cohort of functional MR and the issue of gender-related outcome disparities following MV repair should, therefore, be addressed carefully by future research. A recent study investigated cost-effectiveness of transcatheter MV repair and optimal medical treatment as compared to optimal medical treatment alone in patients with severe secondary MR. Interestingly, females seemed to benefit more from transcatheter treatment as measured in costs per gained quality-adjusted life year (QALY). Similar to TAVR, this may be attributed to a longer general life expectancy of women [44].

### Mitral stenosis

In industrialized countries the incidence of rheumatic valve disease has seen a significant decline over the past decades. Therefore, physicians nowadays predominantly encounter degenerative rather than rheumatic mitral valve stenosis (MS). Regarding the latter, females show a greater disease prevalence [45]; however, they experience favorable outcomes compared to men when treated by percutaneous balloon valvuloplasty [45]. Female gender has been linked to the presence of mitral annulus calcification (MAC), which represents the major anatomical correlate of degenerative MS [46]. Hence, women are also more often affected by non-rheumatic MS [47], a condition with a 5-year mortality rate of over 50%, irrespective of sex [47]. In contrast to rheumatic MS, calcification mainly involves the base of leaflets without any associated commissural fusion, which poses unique challenges to the invasive management of degenerative MS. Surgical mitral valve replacement has traditionally been the treatment of choice for patients with severe degenerative MS, as percutaneous/surgical commissurotomy lacks feasibility here [48]; however, affected patients are usually older with numerous comorbidities and thus a substantial perioperative risk. The unmet need for minimally invasive treatment options of degenerative MS has recently led to the introduction of transcatheter mitral valve devices for severe MAC and failed mitral bioprosthetic valves/annuloplasty rings [49]. Given the higher prevalence of degenerative MS among women, future research should focus on sex-related characteristics in clinical presentation and outcomes among patients undergoing respective interventions.

## Tricuspid regurgitation

Tricuspid regurgitation (TR) predominantly affects women [50] and occurs secondary as a consequence of pressure and/or volume overload followed by annular dilation in the majority of cases, whereas primary TR is rare [51]. Longstanding right ventricular (RV) volume overload due to chronic TR was shown to cause irreversible RV myocardial damage [52], which may significantly worsen the prognosis of patients [53]. Thus, there is an ongoing trend towards an increased performance of tricuspid valve (TV) interventions [54], especially with new transcatheter-based options [55]. Current guidelines recommend performing simultaneous TV intervention with left heart surgery in cases of severe TR or if there are signs of recent right heart failure or annular dilation. If there is no need for left heart surgery, TR should only be addressed if the patient is markedly symptomatic and the window of opportunity has not already closed due to severe LV or RV dysfunction or significant pulmonary hypertension [6]. Isolated surgical treatment of functional TR is associated with high operative morbidity and mortality for both sexes [56]. Significant reduction of TR severity and heart failure symptoms at 6 months was reported in patients undergoing transcatheter edge to edge repair for isolated TR or combined TR/MR [57]; however, explicit reports on gender differences with respect to TV interventions are still lacking.

## Conclusion

There are important sex-related differences regarding clinical presentation, treatment, and outcomes of patients suffering from valvular heart disease. Females present with a distinct risk profile, which poses unique challenges for the invasive treatment of the diseased valve. In general, women face equal or even better long-term survival after surgical/transcatheter treatment, most likely owing to a longer life expectancy. Implementation of sex-specific treatment criteria should be encouraged in order to guarantee timely referral to treatment. With new minimally invasive treatment options on the horizon, future research should emphasize sex-related differences since both sexes benefit from a tailored management with respect to the timing of intervention and treatment modality.

## Abbreviations

AS: Aortic stenosis
AVC: Aortic valve calcification
LV: Left ventricle
LVRR: Left ventricular reverse remodelling
MR: Mitral regurgitation
MV: Mitral valve
RV: Right ventricle
SAVR: Surgical aortic valve replacement
TAVR: Transcatheter aortic valve replacement
TR: Tricuspid regurgitation
TV: Tricuspid valve
